# A retrospective study of structural brain lesions identified by magnetic resonance imaging in 114 cats with neurological signs

**DOI:** 10.14202/vetworld.2023.1871-1879

**Published:** 2023-09-17

**Authors:** Kreevith Prompinichpong, Naris Thengchaisri, Nirut Suwanna, Bordin Tiraphut, Wutthiwong Theerapan, Jörg M. Steiner, Panpicha Sattasathuchana

**Affiliations:** 1 Department of Companion Animal Clinical Sciences, Faculty of Veterinary Medicine, Kasetsart University, Bangkok, 10900, Thailand; 2 Kasetsart University Veterinary Teaching Hospital, Kasetsart University, Bangkok, 10900, Thailand; 3 Department of Small Animal Clinical Sciences, Gastrointestinal Laboratory, School of Veterinary Medicine and Biomedical Sciences, Texas A&M University, College Station, Texas, 77843, USA

**Keywords:** brainstem, cerebellum, cerebrum, feline, seizure

## Abstract

**Background and Aim:**

Magnetic resonance imaging (MRI) has been widely used as a non-invasive modality to evaluate neurological organ structures. However, brain MRI studies in cats with neurological signs are limited. This study evaluated the association between patient characteristics, neurological signs, and brain lesion locations identified by MRI. Blood profiles of cats with presumptive inflammatory and structural brain lesions were also determined.

**Materials and Methods:**

Medical records of 114 cats that underwent brain MRI were retrospectively reviewed. Cats were categorized into five groups based on the location of their lesion: Cerebrum, brainstem, cerebellum, multifocal, and non-structural. Patient characteristics, neurological signs, and hematological profiles were obtained from their medical records. Disease classification was categorized based on their etiologies. Associations were determined using Fisher’s exact test. Blood parameters were compared using the Kruskal–Wallis test.

**Results:**

A total of 114 cats met the inclusion criteria. Lesions were identified in the cerebrum (21.1%), brainstem (8.8%), cerebellum (6.1%), multifocal (39.5%), and non-structural (24.6%) of the cats. Common neurological signs included seizure activity (56.1%), cerebellar signs (41.2%), and anisocoria (25.4%). The most common brain abnormality was inflammation (40.4%). There was no significant difference in hematological profiles between cats with presumptive inflammatory and non-inflammatory brain lesions. Neutrophils, platelets, total protein, and globulin concentrations were higher in cats with structural brain lesions.

**Conclusion:**

The most common neurological signs and brain disease category were seizure activity and inflammation, respectively. However, the hematological profile did not predict inflammatory and structural brain lesions.

## Introduction

The brain is an extremely complex organ with three main compartments: Cerebrum, brainstem, and cerebellum [1, 2]. The cerebrum is necessary for controlling motor and sensory pathways, consciousness, behavior, and memory [3], and cerebral disorders are the most common cause of seizures, visual deficits, mental changes, facial paralysis, circling, and head-turning [4]. The brainstem is the posterior part of the brain connecting the cerebrum and cerebellum to the spinal cord. This structure regulates functions of the autonomic nervous system such as cardiorespiratory, auditory, and temperature control [5–7]. Cranial nerve dysfunction (cranial nerves 3–12) is a disorder arising from brainstem disease [4]. The major function of the cerebellum is to control the coordination of voluntary movement [8]. Cerebellar dysfunction can cause balance disorders and incoordination resulting in ataxia, uncoordinated movement, and nystagmus as a part of the vestibulocerebellar system [5].

DAMNIT-V and magnetic resonance imaging (MRI) are modalities for classifying brain diseases [9–11]. DAMNIT-V is used to evaluate the etiology of abnormalities and can be classified as degenerative, anomalous, metabolic, neoplastic, inflammatory, idiopathic, traumatic, and vascular [10]. Magnetic resonance imaging has been widely used as a non-invasive medical procedure to evaluate the structure of the central nervous system, as it provides an anatomical overview and excellent soft-tissue contrast [11]. The accuracy of ante-mortem neurological disease diagnosis in dogs and cats improved significantly after MRI was introduced into small animal practice [12]. To the best of the author’s knowledge, no prior studies have described the correlation between structural neurological lesions on MRI and clinical abnormalities.

The aims of this retrospective study were: (1) To determine the association between patient characteristics and MRI lesion location, (2) to determine the association between neurological signs and MRI brain lesion location, (3) to classify intracranial diseases of cats using the DAMNIT-V method based on brain lesion location, (4) to compare blood profiles of cats with the presence or absence of inflammatory lesions based on brain MRI, and (5) to compare patient blood profiles with the presence or absence of structural brain lesions on MRI.

## Materials and Methods

### Ethical approval

The study was approved by the Institutional Animal Care and Use Committee (IACUC) of Kasetsart University (ACKU65-VET-057) and found to be in accordance with the guidelines of animal care and use from the Ethical Review Board of the National Research Council of Thailand for the conduct of scientific research. The committee approved and permitted the animal care and use to be conducted as stated in the animal use protocol for this research study. The study was conducted in compliance with the ARRIVE guidelines.

### Study period and location

The research was conducted from January 1, 2018, to December 31, 2021. A retrospective review was undertaken of the medical records of 127 cats that had undergone a brain MRI at Kasetsart University Veterinary Teaching Hospital, Bangkhen campus.

### Case selection

The cats were categorized into five groups based on brain lesion location as determined by MRI: cerebrum, brainstem, cerebellum, multifocal, and non-structural. The multifocal brain lesion group was defined as having a lesion in all brain areas (cerebrum, brainstem, and cerebellum). In total, 13 cats were excluded because of incomplete records (n = 8) or lesions in two brain locations (n = 5). A clinical diagnosis was established based on medical history, hematological and biochemical parameters, neurological examination, MRI findings, cerebrospinal fluid (CSF) analysis, and histopathological findings where available. Hematological and biochemical parameters were assessed using an automated hematology analyzer (Sysmex XN-1000™ Hematology Analyzer, Sysmex, IL, USA) and an automated chemistry analyzer (IL Lab 650 chemistry system, Diamond Diagnostics, MA, USA), respectively. Feline leukemia virus (FeLV) and feline immunodeficiency virus (FIV) testing were performed and recorded using a commercially available FeLV-FIV rapid immunochromatography test assay (Witness^®^, Lyon Cedex, France).

### Data collection

Signalments, clinical signs, neurological signs, results from hematological, serum biochemical, and CSF analyses, brain lesion locations, and the presence of T2-weighted hyperintensity or post-contrast T1-weighted enhancement were obtained from the medical records. Neurological abnormalities were extracted from each animal’s medical records and included seizure activity, cerebellar signs (dysmetria, intention tremor, or cerebellar ataxia), anisocoria, nystagmus, blindness, mental change, circling, tetraparesis, behavioral changes, head-turning, facial paralysis, falling, central vestibular signs, hemiparesis, or head pressing. Disease classification was categorized using the DAMNIT-V scheme based on clinical diagnosis.

### Magnetic resonance imaging

**Figure-1:**
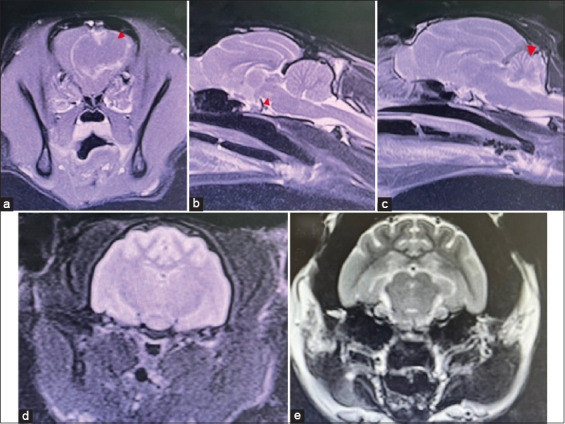
Magnetic resonance imaging of cats with brain lesions according to location. (a) Cerebral neoplasia: The transverse T1-weighted post-contrast image shows mass effect in the left frontal lobe (red arrowhead); (b) brainstem inflammation: The sagittal T2-weighted image of the brainstem shows hyperintensity at cranial brainstem (red arrowhead); (c) cerebellar hypoplasia: The sagittal T2-weighted image of cerebellum shows a decreased volume (red arrow); (d) multifocal brain lesion: The transverse T2 fluid-attenuated inversion image shows inflammatory lesion in cerebrum and brainstem; and (e) non-structural brain lesion: The transverse T2-weighted image shows a normal appearance and intensity of the brain parenchyma.

**Figure-2:**
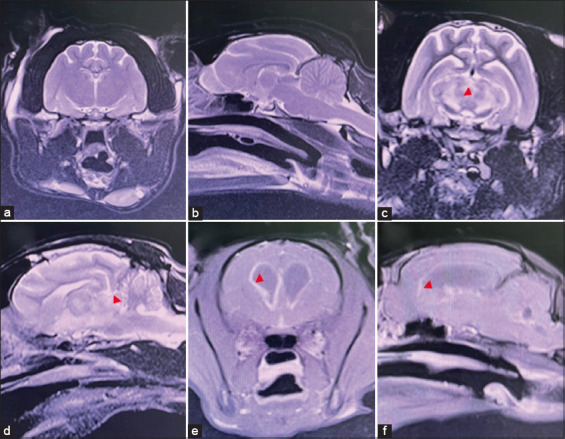
Magnetic resonance imaging of cats with and without presumptive inflammatory brain lesions. (a) transverse T2-weighted image show normal appearance; (b) sagittal T2-weighted image show normal appearance; (c) transverse T2-weighted image shows inflammatory brain lesion; (d) sagittal T2-weighted image shows inflammatory brain lesion; (e) transverse T1-weighted post-contrast image shows the hydrocephalus; and (f) sagittal T1-weighted post-contrast image shows the hydrocephalus. The abnormal lesions are indicated by red arrowheads.

Magnetic resonance imaging was performed using a 1.5 Tesla MRI system (MAGNETOM ESSENZA, Siemens AG, Erlangen, Germany). Structural brain lesions (cerebrum, brainstem, cerebellum, or multifocal) and non-structural brain lesions were identified using MRI (Figure-1). Sagittal, dorsal, T2-weighted, transverse fluid-attenuated inversion recovery, T1-weighted images, and transverse T1-weighted images, after contrast medium administration, were evaluated. Gadoterate meglumine (Dotarem®, Guerbet LLC, New Jersey, USA) was used as a contrast medium to establish the diagnosis and differentiation between inflammatory and non-inflammatory intracranial pathologies. All MRI images were reviewed by a radiological clinician with 10 years of experience reading MRIs and certified by the Board of Veterinary Surgeons with a sub-specialty in veterinary diagnostic imaging. Cats with T2-weighted hyperintensity or T1-weighted post-contrast enhancement on MRI were presumed to have inflammatory brain disease (Figure-2). Abnormalities observed on MRI were categorized according to the DAMNIT-V criteria.

### Statistical analysis

Statistical analyses were performed using a commercially available software package (GraphPad Prism 9.3.1, GraphPad Software Inc., San Diego, CA). Normality testing was performed using the Shapiro–Wilk test. Kruskal–Wallis and *post hoc* tests were used to compare continuous data (age and hematological and biochemistry parameters). Fisher’s exact test was used to determine the association between brain lesion location and signalment, neurological signs, or DAMNIT-V classification. p < 0.05 was considered statistically significant.

## Results

Of the 114 cats that met inclusion criteria, 72 (63.2%) were male and 42 (36.8%) were female. The median (range) age of the cats at the time of diagnosis was 24 (4–204) months. Hair length was classified as short-haired or long-haired, with 96 (84.2%) and 18 (15.8%) cats meeting those criteria, respectively. There were 75 (65.8%) brachycephalic and 39 (34.2%) non-brachycephalic breeds. Of the 114 cats, a structural brain lesion was identified in 86 (75.4%), and of those 86 lesions, 24 were in the cerebrum (21.1%), 10 in the brainstem (8.8%), 7 in the cerebellum (6.1%), and 45 multifocal (39.5%). Of the 114 cats, 64 (56%) had a record of FeLV antigen and FIV antibody test results: FeLV positive (n = 7; 10.9%), FIV positive (n = 3; 4.7%), both FeLV and FIV positive (n = 1; 1.6%), and both FeLV and FIV negative (n = 53; 82.8%).

Each brain lesion location is presented in Table-1. The groups had significant age differences (p = 0.0253) and brachycephalic breed (p = 0.0149). The demographic characteristics, including sex (p = 0.7210) or hair length (p = 0.2883), were not significantly different among the various groups. There was an association between brachycephalic cats and cats with a non-cerebral lesion (p = 0.0145).

**Table-1:** Patient characteristics according to brain lesion locations.

Signalment	Brain lesion locations					P-value

	Cerebrum (n = 24)	Brainstem (n = 10)	Cerebellum (n = 7)	Multifocal (n = 45)	Non-structural (n = 28)	
Median Age (range; months)	48	32.5	9	12	24	0.0253
	(4–204)	(8–144)	(5–180)	(4–168)	(5–156)	
Sex						0.7210
Male	14 (58.33%)	6 (60%)	4 (57.14%)	31 (71.11%)	16 (57.14%)	
Female	10 (41.67%)	4 (40%)	3 (42.86%)	13 (28.89%)	12 (42.86%)	
Hair length						0.2883
Short-haired	23 (95.83%)	8 (80%)	6 (85.71%)	38 (84.44%)	21 (75%)	
Long-haired	1 (4.17%)	2 (20%)	1 (14.29%)	7 (15.56%)	7 (25%)	
Brachycephalic breed	3 (12.5%) OR (95%CI) = 0.21 (0.06–0.74)	1 (10%)	3 (42.86%)	19 (42.22%)	13 (46.43%)	0.0149

OR=odds ratio; 95% CI=95% confidence Interval

Neurological signs based on brain lesion location consisted of seizures (n = 64; 56.1%), cerebellar signs (n = 47; 41.2%), anisocoria (n = 26; 25.4%), nystagmus (n = 23; 20.2%), blindness (n = 15; 13.2%), mental change (n = 13; 11.4%), circling (n = 12; 10.5%), tetraparesis (n = 12; 10.5%), behavioral change (n = 6; 5.3%), head turn (n = 5; 4.4%), facial paralysis (n = 3; 2.6%), falling (n = 3; 2.6%), central vestibular signs (n = 2; 1.8%), hemiparesis (n = 2; 1.8%), and head pressing (n = 1; 0.9%) (Table-2). There were associations between seizure activity and cats with non-brainstem lesions (p = 0.0005) and cats with non-structural brain lesions (p = 0.0005). Associations were observed between cerebellar signs and various conditions: Non-cerebral lesions (p = 0.0343), cerebellar lesions (p = 0.0190), or multifocal brain lesions (p = 0.0190). An association was identified between anisocoria and cats with structural brain lesions (p = 0.0355). In addition, circling was associated with cerebral lesions (p = 0.0135), and central vestibular signs were associated with cerebellar lesions (p = 0.0288). Moreover, facial paralysis was found to be associated with cerebellar lesions (p = 0.0461).

**Table-2:** Neurological signs according to brain lesion locations.

Neurological signs	Brain lesion locations				

	Cerebrum (n = 24)	Brainstem (n = 10)	Cerebellum (n = 7)	Multifocal (n = 45)	Non-structural (n = 28)
Seizure (n = 64)	16 (66.67%)	0 (0%) OR (95% CI) = 0.06 (0.01–0.35)	2 (28.57%)	22 (48.89%)	24 (85.71%) OR (95% CI) = 5.73 (2.02–14.58)
Cerebellar signs (n = 47)	5 (20.83%) OR (95% CI) = 0.30 (0.12–0.82)	3 (30%)	6 (85.71%) OR (95% CI) = 9.66 (1.46–112.3)	25 (55.56%) OR (95% CI) = 2.67 (1.24–5.54)	8 (28.57%)
Anisocoria (n = 26)	7 (29.17%)	4 (40%)	1 (14.29%)	12 (26.67%)	2 (7.14%) OR (95% CI) = 0.20 (0.04–0.78)
Nystagmus (n = 23)	4 (16.67%)	2 (20%)	1 (14.29%)	9 (20%)	7 (25%)
Blindness (n = 15)	6 (25%)	1 (10%)	0 (0%)	7 (15.56%)	1 (3.57%)
Mental changes (n = 13)	5 (20.83%)	1 (10%)	1 (14.29%)	5 (11.11%)	1 (3.57%)
Circling (n = 12)	6 (25%) OR (95% CI) = 4.47 (1.47–13.49)	2 (20%)	1 (10%)	3 (6.67%)	0 (0%)
Tetraparesis (n = 12)	1 (4.17%)	2 (20%)	0 (0%)	8 (17.78%)	1 (3.57%)
Behavioral change (n = 6)	3 (12.50%)	1 (10%)	0 (0%)	2 (4.44%)	0 (0%)
Head turn (n = 5)	1 (4.17%)	1 (10%)	0 (0%)	3 (6.67%)	0 (0%)
Facial paralysis (n = 3)	1 (4.17%)	0 (0%)	1 (14.29%) OR (95% CI) = 10.10 (1.53–53.94)	0 (0%)	1 (3.57%)
Falling (n = 3)	1 (4.17%)	0 (0%)	0 (0%)	2 (4.65%)	0 (0%)
Central vestibular disorders (n = 2)	0 (0%)	0 (0%)	1 (14.29%) OR (95% CI) = 15.29 (2.04–102.6)	1 (2.22%)	0 (0%)
Hemiparesis (n = 2)	1 (4.17%)	0 (0%)	0 (0%)	1 (2.22%)	0 (0%)
Head pressing (n = 1)	0 (0%)	0 (0%)	0 (0%)	1 (2.22%)	0 (0%)

OR=odds ratio; 95% CI=95% confidence Interval

Brain diseases of the cats in this study were classified using the DAMNIT-V scheme resulting in classification as degenerative (n = 5; 4.4%), anomalous (n = 19; 16.7%), metabolic (n = 2; 1.8%), neoplastic (n = 11; 9.7%), inflammatory (n = 46; 40.4%), idiopathic (n = 24; 21.1%), traumatic (n = 6; 5.3%), or vascular (n = 1; 0.9%) (Table-3). Cerebral lesions were significantly associated with degenerative brain disease (p < 0.0001). Anomalous brain disease was associated with various conditions: structural brain lesions (p = 0.0139), cerebellar lesions (p = 0.0088), and multifocal brain lesions (P = 0.072). Structural brain lesions (p < 0.0001) or multifocal brain lesions (p = 0.0109) were also associated with presumptive inflammatory brain disease. However, no association was found between retrovirus infection and presumptive inflammatory brain disease (p = 0.3311). Traumatic brain disease was associated with cerebral lesions (p = 0.0127). Idiopathic epilepsy was associated with non-cerebral brain lesions (p = 0.0137), non-multifocal brain lesions (p < 0.0001), and non-structural brain lesions (p < 0.0001).

**Table-3:** DAMNIT-V diagnosis according to brain lesion locations.

DAMNIT-V	Brain lesion location				

	Cerebrum (n = 24)	Brainstem (n = 10)	Cerebellum (n = 7)	Multifocal (n = 45)	Non-structural (n = 28)
Degenerative (n = 5)	3 (12.5%) OR (95% CI) = 5.39 (1.34–22.21)	1 (10%)	0 (0%)	1 (2.22%)	0 (0%)
Anomaly (n = 19)	2 (8.33%)	0 (0%)	4 (57.14%) OR (95% CI) = 7.27 (1.83–25.09)	13 (28.89%) OR (95% CI) = 3.88 (1.47–9.80)	0 (0%) OR (95% CI) = 0.12 (0.01–0.75)
Metabolic (n = 2)	0 (0%)	0 (0%)	0 (0%)	0 (0%)	2 (7.14%)
Neoplastic (n = 11)	3 (12.5%)	2 (20%)	1 (14.29%)	5 (11.11%)	0 (0%)
Inflammatory (n = 46)	11 (45.83%)	7 (70%)	1 (14.29%)	25 (55.56%) OR (95% CI) = 2.86 (1.32–5.97)	2 (7.14%) OR (95% CI) = 0.07 (0.02–0.31)
Idiopathic (n = 24)	0 (0%) OR (95% CI) = 0.11 (0.01–0.67)	0 (0%)	0 (0%)	0 (0%)*** OR (95% CI) = 0.04 (0–0.24)	24 (85.71%) OR (95% CI) = 435 (54.99–4,478)
Traumatic (n = 6)	4 (16.67%) OR (95% CI) = 7.06 (1.62–27.70)	0 (0%)	1 (14.29%)	1 (2.22%)	0 (0%)
Vascular (n = 1)	1 (4.17%)	0 (0%)	0 (0%)	0 (0%)	0 (0%)

OR=odds ratio; 95% CI=95% confidence Interval

Hematological and serum chemistry profiles of cats with and without presumptive inflammatory brain lesions are noted in Table-4. No significant differences were observed in the hematological or biochemical parameters between cats with presumptive inflammatory brain lesions and those with non-inflammatory brain lesions (p > 0.05).

**Table-4:** Comparison of hematological and serum chemistry parameters between presence and absence of inflammatory lesions on MRI.

Parameters (median (range))	Reference interval	Presence or absence of inflammatory lesion		P-value

		Non-inflammatory (n = 64)	Inflammatory (n = 50)	
Hematocrit	30**–**45%	35.1 (21.8**–**49.7)	35.5 (22.9**–**55)	0.9863
Red blood cell	5-10 x10^6^/μL	8.33 (4.1**–**12.12)	7.89 (4.97**–**12.28)	0.5251
Mean corpuscular volume	39**–**55 fL	42.47 (31.1**–**60.9)	43.95 (4.1**–**62.17)	0.8416
Mean corpuscular hemoglobin concentration	30**–**36 gm%	35.4 (27.5**–**40.2)	34.03 (30.19**–**40.86)	0.3624
White blood cell count	5,000**–**19,000 cells/μL	10,860 (5,570**–**23,980)	11,890 (2,630**–**36,600)	0.8327
Neutrophil count	2,500**–**12,500 cells/μL	7,981 (567.36**–**18,944.2)	8,274.5 (1,670**–**34,770)	0.8371
Lymphocyte count	1,500**–**7,000 cells/μL	1,973.96 (215.6**–**19,800)	1,891 (168**–**19,430)	0.9545
Monocyte count	0**–**850 cells/μL	216.23 (0**–**1,092)	156.76 (0**–**1,580)	0.3441
Eosinophil count	0**–**750 cells/μL	364.1 (0**–**2,379)	175.3 (0**–**3,276)	0.0703
Basophil count	Rare	0 (0**–**170.5)	0 (0**–**240)	0.2054
Platelet count	200**–**800 x10^3^/μL	209 (10**–**170.5)	228 (22.7**–**633)	0.9023
Blood urea nitrogen concentration	15**–**34 mg%	23.17 (6**–**43)	21 (12**–**45)	0.4696
Serum creatinine concentration	1.0**–**2.2 mg%	1.3 (0.6**–**2.6)	1.31 (0.4**–**2.5)	0.7884
Alanine transaminase activity	28**–**76 IU/L	51.5 (17**–**636)	54 (27**–**730)	0.3316
Total protein concentration	5.8**–**7.8 gm%	7.2 (2.5**–**11.4)	7.26 (5.5**–**9.6)	0.4454
Albumin concentration	2.6**–**4.2 gm%	3.38 (2.7**–**8.1)	3.4 (2.4**–**4.3)	0.6591
Globulin concentration	2.6**–**5.1 gm%	3.8 (2.4**–**8.4)	3.9 (2.4**–**6.9)	0.8227

Hematological and chemistry profiles of cats with and without structural brain lesions are noted in Table-5. The neutrophil count was significantly increased in cats with structural brain lesions compared to those with non-structural brain lesions (p = 0.0329). Similarly, the platelet count was significantly higher in cats with structural brain lesions than those with non-structural brain lesions (p = 0.0270). Moreover, the serum total protein concentration was higher in cats with structural brain lesions than in those with non-structural brain lesions (p = 0.0088). Finally, the serum globulin concentration was significantly increased in cats with structural brain lesions compared to those with non-structural brain lesions (p = 0.0142).

**Table-5:** Comparison of hematological and serum chemistry parameters between cats with or without structural lesions on MRI.

Parameter [median (range)]	Reference	Presence or absence of structural lesion		P-value

		Non-structural (n = 28)	Structural (n = 86)	
Hematocrit	30**–**45%	36.5 (23.8–49)	34.8 (21.8–55)	0.2027
Red blood cell	5–10 x10^6^/μL	8.78 (5.32–12.12)	7.99 (4.1–12.28)	0.1647
Mean corpuscular volume	39**–**55 fL	42.75 (4.16–60.9)	43.09 (26.6–62.17)	0.8771
Mean corpuscular hemoglobin concentration	30**–**36 gm%	33.4 (28.2–38.37)	35.29 (27.5–40.86)	0.0852
White blood cell count	5,000**–**19,000 cells/μL	9,710 (5,570–36,600)	11,850 (2,630–36,400)	0.058
Neutrophil count	2,500**–**12,500 cells/μL	6,044.7 (567.36–34,770)	8,515.2 (1,851.52–24,388)	0.0329
Lymphocyte count	1,500**–**7,000 cells/μL	1,795.2 (215.6–6,429.5)	1,973.96 (168–19,800)	0.958
Monocyte count	0–850 cells/μL	152.74 (0–1,580)	222.73 (0–1,092)	0.2668
Eosinophil count	0–750 cells/μL	344.05 (0–2,212)	304.07 (0–3,276)	0.5486
Basophil count	Rare	0 (0–120.6)	0 (0–240)	0.2473
Platelet count	200–800 x10^3^≥/μL	177 (22.7–438)	239 (10–633)	0.027
Blood urea nitrogen concentration	15–34 mg%	23 (10–38.8)	22 (6–45)	0.9108
Serum creatinine concentration	1.0–2.2 mg%	1.31 (0.6–2.17)	1.3 (0.4–2.6)	0.742
Alanine transaminase activity	28–76 IU/L	57 (27–730)	53 (17–636)	0.4709
Total protein concentration	5.8–7.8 gm%	7 (5.8–8.3)	7.31 (5.5–11.4)	0.0088
Albumin concentration	2.6–4.2 gm%	3.39 (2.7–4.2)	3.38 (2.4–4.3)	0.7815
Globulin concentration	2.6–5.1 gm%	3.7 (2.6–5.3)	3.9 (2.4–8.4)	0.0142

## Discussion

To the best of our knowledge, this study presents the first report of descriptive neurological signs in cats undergoing MRI. A retrospective review of 114 cats over 4 years found the most common signs to include seizures (n = 64), cerebellar signs (n = 47), and anisocoria (n = 26). Common causes of brain disease were presumptive inflammation (n = 46), idiopathic lesions (n = 24), and anomalous diseases (n = 19). Cats with structural brain lesions had significantly higher neutrophil and platelet counts and total protein and globulin concentrations than those with non-structural brain lesions.

Non-cerebral lesions have previously been associated with brachycephalic breeds. This may be explained by the anatomical structure of brachycephalic animals. Occipital bone malformation in brachycephalic breeds causes compression of the caudal fossa which is adjacent to the cerebellum and brainstem, but not the cerebrum [13]. Accordingly, cerebral lesions were not commonly observed in brachycephalic cats in this study.

The most common neurological sign observed was seizure activity. Associations between seizure activity and non-brainstem and non-structural brain lesions were also identified. In contrast, the previous studies by Matiasek *et al*. [14] have reported that brainstem lesions were not associated with seizure activity in domestic animals. This discrepancy may partly be explained by the cerebrum being the only seizure center identified in domestic animals [15]. The high prevalence of seizure activity in cats without structural brain lesions (21.1%) may also suggest a high prevalence of idiopathic epilepsy in cats. This notion is further supported by a prior report that noted a prevalence of feline idiopathic epilepsy as high as 33% [16]. Notably, a lower prevalence of idiopathic epilepsy in dogs (0.6%) has been reported [17]. Whether cats are specifically predisposed to idiopathic epilepsy requires further investigation.

Cerebellar signs (41.2%) included cerebellar ataxia, intention tremor, and decerebellate rigidity [18]. In a prior study, cerebellar signs were commonly found in cats with cerebellar and multifocal brain lesions [19]. Cats with multifocal brain lesions included in this study had cerebral, brainstem, and cerebellar lesions. This would explain why cats with multifocal brain lesions would also have cerebellar signs.

Anisocoria (25.4%) was the second-most common neurological sign observed in this study, and to the best of our knowledge, this is the first time that this has been reported in cats. Anisocoria was observed in cats with structural brain lesions. This may be explained by miosis being associated with forebrain and midbrain lesions. Lesions of the forebrain and midbrain lead to a loss of sympathetic function or upper motor neuron inhibition of the oculomotor nerve, causing pupillary constriction in the affected eye [20].

Central vestibular signs (1.8%) were observed in cats with cerebellar lesions. This may be explained by the fact that the blood supply to the brainstem, cerebellum, and inner ear is derived from the vertebrobasilar system [21]. Interruption of the vestibular nuclei-archicerebellar loop may cause central paroxysmal positional vertigo [22]. Another study of vestibulocerebellar syndrome in three dogs with presumed cerebellar hypoplasia noted that vestibular syndrome is related to a disease process affecting the vestibulocochlear nerve, medulla oblongata, brainstem, thalamus, and cerebellum [22].

The DAMNIT-V scheme found the most common etiologies to be presumptive inflammatory (40.4%), idiopathic (21.1%), and anomalous (16.7%). Degenerative brain disease (4.4%) was the most common etiology in cats with cerebral lesions. Similar to humans, this may be explained by the cerebral degenerative changes that occur during aging [23].

Anomalous brain disease (16.7%) was the most commonly identified etiology in cats with cerebellar lesions. This may be caused by early prenatal feline parvovirus infection that has been widely reported in cats with cerebellar lesions [24]. Parvovirus affects the development of the brain, especially the cerebellum. Parvovirus infection in cats in Thailand is commonly found in clinical practice (58.7%) [25]. Therefore, the cerebellar anomaly observed in this study may have been caused by an *in utero* parvoviral infection. Furthermore, the anomaly was associated with cats having multifocal brain lesions. A structural abnormality can occur during brain development, causing abnormalities throughout the brain.

Presumptive inflammatory brain disease was observed in cats with multifocal brain lesions. Progressive stages of inflammatory brain disease have been reported with perivascular inflammation of the entire brain [26]. In addition, cats with multifocal brain lesions showed progressive clinical signs at the time of diagnosis in the present study. Notably, there was no association between retroviral infection and presumptive inflammatory brain disease in the present study. This is in contrast to another study that found FeLV in the spinal cord of cats causing structural neurological damage [27]. The discordance of these results may be explained by the limitations of this being a retrospective study, and FeLV and FIV infection were not tested in all enrolled cats. Further studies should be conducted to identify the relationship between cats with retroviral infection and neurological signs. In this study, two cats with non-structural lesions on MRI were classified as presumptive inflammatory brain disease because CSF analysis revealed the presence of inflammation.

Traumatic brain disease (5.3%) was the most common etiology found in cats with cerebral lesions. This may be explained by the cerebrum being the largest, and, more importantly, most superficially located part of the brain. Brain contusions generally affect the cerebral gyri and extend through the cortex [28]. Therefore, it is not surprising that the cerebrum was the area most affected by trauma.

The diagnosis of idiopathic epilepsy depended on the absence of structural abnormalities on MRI evaluation of seizure activity. The prevalence of idiopathic epilepsy in cats in the present study was 21.1%. This is much greater than the prevalence of idiopathic epilepsy in dogs (6.8%) in a previous report by Loncarica *et al*. [29]. The higher prevalence of seizure activity in cats may be explained by reports of a genetic predisposition of cats to idiopathic epilepsy [16, 30].

There was no significant difference in the hematological and biochemical parameters between cats with presumptive inflammatory brain lesions and those with non-inflammatory brain lesions. This is similar to reports in dogs and other reports in cats [31, 32]. Cats with meningoencephalomyelitis of unknown origin have been reported to have hematological and biochemical parameters within normal reference ranges [31]. Furthermore, dogs with necrotizing meningoencephalitis have been reported to present with normal hematological and biochemical parameters [32]. Thus, these parameters may not be useful for predicting presumptive inflammatory brain lesions in cats with neurological signs.

To the best of our knowledge, this is the first report comparing hematological and biochemical parameters between cats with structural and non-structural brain lesions. Neutrophil and platelet counts and total protein and globulin concentrations were increased in cats with structural brain lesions. However, the clinical significance of these findings for the diagnosis of structural brain lesions remains unclear. Further studies should investigate the causes and predictive values of blood profiles for structural brain disease.

A limitation of this study was its retrospective nature, thus increasing the possibility of a Type 2 error. The number of cats with cerebellar and brainstem lesions was small compared to other groups. However, this study did note the prevalence of brain lesions that primarily affected multiple brain locations. A second limitation was that histopathological diagnoses were unavailable to confirm a final diagnosis in some cats, as ante-mortem examinations were not routinely performed in veterinary clinical practice. Further studies should investigate non-invasive diagnostic tools for obtaining histological samples from cats with neurological disease.

Cerebrospinal fluid collection and analysis were performed in 26.3% of the cats enrolled in this study. The reason for not performing CSF collection was the potential for cerebellar herniation. Contraindications to CSF collection through the cerebellomedullary cistern are atlantoaxial subluxation, Chiari-like malformation, and the risk of cerebellar herniation through the foramen magnum [33]. Cats with brain herniation have a significantly lower level of consciousness in their Modified Glasgow Coma Scale score [34]. The lack of CSF analysis in all cases was thus a limitation of this study.

## Conclusion

Magnetic resonance imaging was shown to be a useful, non-invasive, and diagnostic modality in cats with suspected brain disease. The most common neurological signs were seizure activity, cerebellar signs, and anisocoria. The predominant causes of brain disease were inflammatory and idiopathic brain lesions. Hematologic and blood chemistry profiles may not be useful for these diagnoses.

## Data Availability

The datasets used and analyzed during the study can be available from the corresponding author on a reasonable request.

## Authors’ Contributions

KP: Data collection, data analysis, data interpretation, literature search, and drafting of the manuscript. NT and JMS: Study design and review of the manuscript. NS, BT, and WT: Interpreted data and reviewed manuscript. PS: Study design, data analysis, data interpretation, and review of the manuscript. All authors have read, reviewed, and approved the final manuscript.
